# Retention of Ag‐specific memory CD4^+^ T cells in the draining lymph node indicates lymphoid tissue resident memory populations

**DOI:** 10.1002/eji.201646681

**Published:** 2017-04-11

**Authors:** Clare L. Marriott, Emma E Dutton, Michio Tomura, David R. Withers

**Affiliations:** ^1^ Institute of Immunology & ImmunotherapyCollege of Medical and Dental SciencesUniversity of BirminghamUK; ^2^ Laboratory of ImmunologyFaculty of PharmacyOsaka‐Ohtani University 3‐11‐1 NishikiorikitaTondabayashi‐cityOsaka prefectureJapan

**Keywords:** Cell trafficking, Lymph nodes, Memory CD4^+^ T cells, *Memory cells*

## Abstract

Several different memory T‐cell populations have now been described based upon surface receptor expression and migratory capabilities. Here we have assessed murine endogenous memory CD4^+^ T cells generated within a draining lymph node and their subsequent migration to other secondary lymphoid tissues. Having established a model response targeting a specific peripheral lymph node, we temporally labelled all the cells within draining lymph node using photoconversion. Tracking of photoconverted and non‐photoconverted Ag‐specific CD4^+^ T cells revealed the rapid establishment of a circulating memory population in all lymph nodes within days of immunisation. Strikingly, a resident memory CD4^+^ T cell population became established in the draining lymph node and persisted for several months in the absence of detectable migration to other lymphoid tissue. These cells most closely resembled effector memory T cells, usually associated with circulation through non‐lymphoid tissue, but here, these cells were retained in the draining lymph node. These data indicate that lymphoid tissue resident memory CD4^+^ T‐cell populations are generated in peripheral lymph nodes following immunisation.

## Introduction

Naïve CD4^+^ T cells recirculate through secondary lymphoid tissue in search of Ag presenting cells displaying cognate peptides in the context of MHCII. With appropriate costimulation, interactions through the TCR induce these naïve CD4^+^ T cells to form a heterogeneous population of effectors cells which ultimately establish multiple memory populations that provide long term protection 1. Seminal early studies identified two populations of memory CD4^+^ T cells on the basis of CD62L and CCR7 expression, molecules that determine the ability of cells to enter lymph nodes (LNs) from the circulation via high endothelial venules 2. Thus cells lacking CD62L and CCR7 were termed effector memory T cells (Tem) and considered to be tissue homing, unable to recirculate through LNs, but able to rapidly generate effector cytokines upon Ag re‐encounter. Those memory cells expressing CD62L and CCR7 were termed central memory T cells (Tcm) and considered able to recirculate through secondary lymphoid tissue, thus providing a means to support rapid secondary responses that generate subsequent waves of effector cells 2. Analysis of endogenous Ag‐specific CD4^+^ T‐cell responses in mice has generated data supporting these initial experiments using human polyclonal cells 3. Building on the initial description of Tem, it became apparent that some memory CD4^+^ T‐cell populations preferentially migrated to non‐lymphoid tissues such as the skin, intestine or lung and then remained at these sites forming tissue resident memory T‐cell populations (Trm) 4, 5, 6, 7.

Relying on surface markers to assess tissue migration has caveats, not least the indirect assessment of migration potential, rather than actual demonstration of trafficking. Carefully controlled recent *in vivo* studies indicate that CCR7 is crucial for naïve but not memory CD4^+^ T‐cell recirculation through lymph nodes, suggesting memory cells can utilise other mechanisms to enter these sites 8. Recent data revealing changes in CD62L expression during a response further argues against reliance on surface markers to categorise migratory populations 9. The advent of new tools to assess cellular migration, particularly the generation of transgenic mice expressing photoconvertible proteins has heralded more sophisticated analyses of T‐cell migration 10, 11, 12. However, a lack of Ag‐specificity in these studies has limited understanding of the exact stage of the response being assessed. Fundamental studies of endogenous CD4^+^ T‐cell populations in mice have established reproducible approaches to track polyclonal Ag‐specific CD4^+^ T cells throughout a response 3, 13, 14, 15 . Here we sought to restrict the draining secondary lymphoid tissue to a specific LN and then assess the memory CD4^+^ T‐cell populations generated here and determine the migration of these cells to other lymphoid tissues. Through assessing an endogenous Ag‐specific response in photoconvertible Kaede transgenic mice, we demonstrated that a locally produced memory CD4^+^ T‐cell population is retained in the draining LN and does not recirculate through other lymphoid tissues, consistent with the establishment of lymphoid tissue resident memory populations.

## Results

### Brachial LN model of 2W1S‐specific response

**Figure 1:**
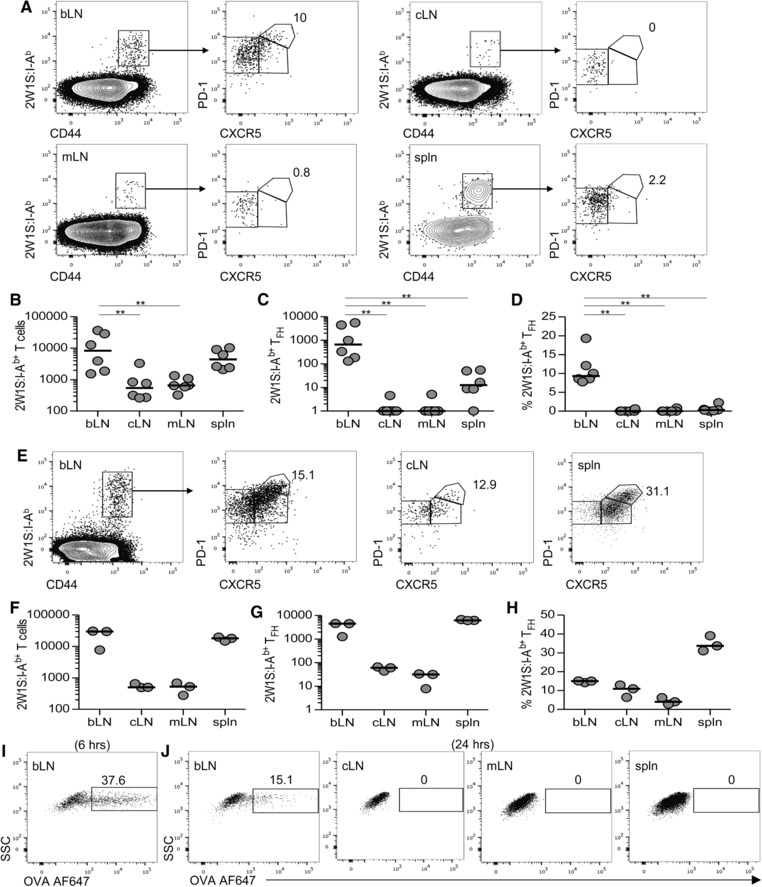
Model to assess 2W1S‐specific CD4^+^ T‐cell response initiated in the draining brachial lymph node. (A–H) C57BL/6 mice were immunised with (A–D) 5 μg 2W1S peptide or (E–H) 20 μg 2W1S peptide precipitated with alum in the right front paw pad, then analysed at 8 dpi. (A) Expression of CXCR5 and PD‐1 on 2W1S:I‐A^b+^ CD4^+^ T cells from the brachial LN (bLN), a pool of contralateral LNs (cLN), mesenteric LNs (mLN) and spleen (spln) was evaluated by flow cytometry, values show percentage of CXCR5^hi^PD‐1^+^ T_FH_ cells. (B–D) The number of (B) 2W1S:I‐A^b+^ CD4^+^ T cells, (C) 2W1S:I‐A^b+^ CD4^+^ T_FH_ cells and (D) percentage of 2W1S:I‐A^b+^ CD4^+^ T_FH_ cells in each tissue were evaluated by flow cytometry. (E) Expression of CXCR5 and PD‐1 on 2W1S:I‐A^b+^ CD4^+^ T cells from bLN, cLN and spleen, values show % T_FH_ cells. (F–H) The number of (F) 2W1S:I‐A^b+^ CD4^+^ T cells, (G) 2W1S:I‐A^b+^ CD4^+^ T_FH_ cells and (H) percentage of 2W1S:I‐A^b+^ CD4^+^ T_FH_ cells in each tissue are shown. (I and J) WT mice were injected with 5 μg Ovalbumin‐Alexa Fluor 647 precipitated with alum in the right paw pad.Expression of Alexa Fluor 647 by MHCII^+^CD11c^+^, B220^−^CD3^−^ cells in draining bLN at (I) 6 h and (J) 24 h post immunisation in the tissue indicated. Flow cytometry plots are representative of 6 (A) and 3 (E, I, J) mice. (B‐D, F–H) Each symbol represents an individual mouse and data are pooled from two (B‐D) or one (F‐H) independent experiment. Values of 0 were assigned a value of 1 for logarithmic scales; bars show medians. Mann–Whitney test: **p < 0.01.

Secondary lymphoid tissues contain a heterogeneous mix of CD44^hi^ CD4^+^ T cells that differ in expression of the surface molecules thought to enable recirculation through lymphoid and non‐lymphoid. Whether all these cells were initially generated within the tissue, trafficked to these sites and/or modified their expression of the surface markers used to phenotype them is often impossible to resolve. Thus, to better understand these populations within LNs, we sought to establish a model where a specific LN was the principal site of initiation for an endogenous Ag‐specific CD4^+^ T‐cell response. This approach would allow us to identify when populations were generated and clarify the movement of memory CD4^+^ T cells to other secondary lymphoid tissue or their retention in the draining LN. Immunisation of the murine paw pad has been used to target a specific peripheral LN, often the popliteal LN 16. We immunised the front paw pad to target the brachial LN (bLN) given its greater size and the scarcity of endogenous CD4^+^ T cells responsive to a given peptide 13, 15. Thus mice were immunised in the front paw pad with 5μg 2W1S peptide precipitated with alum and at 8 days post immunisation (dpi), a robust number of 2W1S‐specific CD44^hi^ CD4^+^ T cells were evident in the draining bLN (Fig. 1A). These cells could be separated into the CXCR5^−^PD‐1^int^, CXCR5^+^PD‐1^int^ and CXCR5^+^PD‐1^hi^ populations previously described 3 with the latter population consistent with the generation of a T follicular helper (T_FH_) cell population (Fig. 1A). Notably, whilst a small number of 2W1S‐specific CD44^hi^ CD4^+^ T cells were also detected in a pool of contralateral LNs (cLN, comprised of axillary, brachial and inguinal LNs from the opposite side of the mouse) and the mesenteric LNs (mLN), the T_FH_ population was absent (Fig. 1A–D). Previous studies have indicated that T_FH_ are LN resident 17, 18 and their detection only in the draining bLN indicates this is the principal site for the initiation of the response. Whilst a larger number of 2W1S‐specific CD4^+^ T cells were detected within the spleen, again the T_FH_ population was largely absent (Fig. 1A–D). Importantly, when mice were immunised with a larger dose of Ag (20 μg peptide precipitated with alum) both a greater of number of 2W1S‐specific CD44^hi^ CD4^+^ T cells but also a clear T_FH_ population was detected in all secondary lymphoid tissues analysed (Fig. 1E–H). These data argue that when larger doses of Ag are given, enhanced dissemination of Ag to other tissues occurs and the response is not limited to a given draining LN. Furthermore, following immunisation with 5 μg Ovalbumin‐Alexa Fluor 647 precipitated in alum, Alexa Fluor 647^+^CD11c^+^MHCII^+^ cells were detected in the draining bLN, but not other secondary lymphoid tissues at 6 and 24 h post immunisation (Fig. 1I and J). Combined these data indicate that immunisation into the paw pad with 5ug Ag precipitated with alum initiated a response within the draining bLN with minimal spread of Ag to other tissues. Thus, these data demonstrate a means of assessing 2W1S‐specific memory CD4^+^ T cells generated within a given peripheral LN.

**Figure 2:**
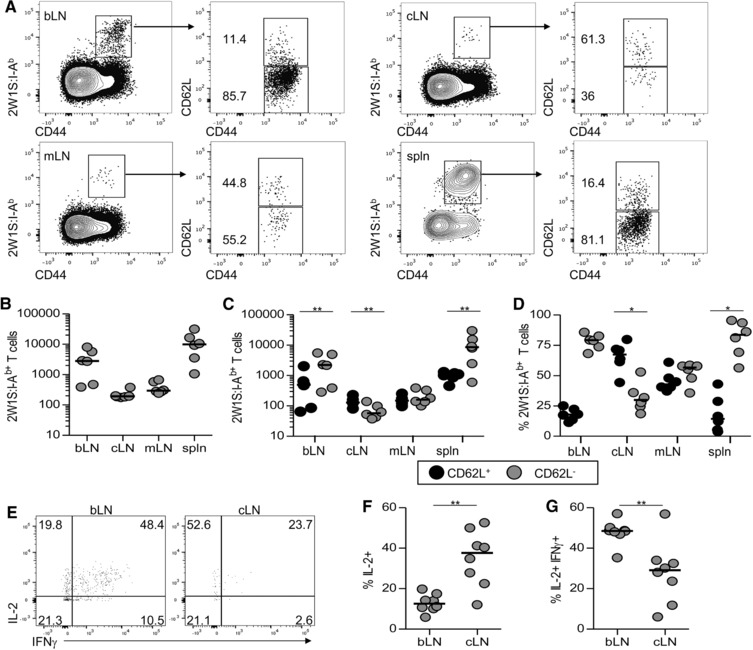
2W1S‐specific Tem cells persist within the draining brachial LN. Memory 2W1S‐specific T cells were assessed at 42 dpi of C57BL/6 mice with 5 μg 2W1S peptide precipitated with alum. (A) Expression of CD62L and CD44 on 2W1S:I‐A^b+^ CD4^+^ T cells from brachial LN (bLN), a pool of contralateral LNs (cLN), mesenteric LNs (mLN) and spleen (spln) was evaluated by flow cytometry. (B) The number of 2W1S:I‐A^b+^ CD4^+^ T cells isolated from different tissues is shown. (C, D) The (C) number and (D) percentage of CD62L^+^ and CD62L^−^ cells amongst 2W1S:I‐A^b+^ CD4^+^ T cells isolated from different tissues is shown. (E) Expression of IL‐2 and IFN‐γ by 2W1S‐specific CD4^+^ T cells following ex vivo stimulation. (F, G) The percentages of (F) IL‐2^+^ and (G) IL‐2^+^IFN‐γ^+^ 2W1S‐specific CD4^+^ T cells in bLN and cLN are shown. (A, E) Flow cytometry plots are representative of 6 (A) and 8 (E) mice. (B–D, F–G) Each symbol represents an individual mouse and data are pooled from two independent experiments; bars show medians. Mann‐Whitney test: *p < 0.01, **p < 0.01.

To assess the distribution of memory 2W1S‐specific CD4^+^ T cells in this model, C57BL/6 mice were immunised with 5 μg 2W1S peptide/alum as described above and then tissues analysed at 42 dpi. A substantial population of 2W1S‐specific CD44^hi^ CD4^+^ T cells were still present within the bLN at this time, whilst a smaller population of 2W1S‐specific CD44^hi^ CD4^+^ T cells were detected within cLN and mLN (Fig. 2A and B). A substantial population of 2W1S‐specific CD44^hi^ CD4^+^ T cells also was present in the spleen (Fig. 2A and B). Expression of CD62L is required for lymphocytes to enter LNs via high endothelial venules and thus CD62L expression, along with CCR7 is thought to be a key marker for discriminating memory cells that recirculate through lymphoid tissue rather than non‐lymphoid tissue 2, 19, 20. The majority of 2W1S‐specific CD44^hi^ CD4^+^ T cells in the cLNs were CD62L^+^ (Fig. 2C and D), whilst in the mLN and spleen, known to not depend on CD62L expression for entry 21, 22, more CD62L^−^ cells were evident (Fig. 2C and D). Notably, the vast majority of 2W1S‐specific CD4^+^ T cells remaining in the draining bLN lacked expression of CD62L, suggesting these cells could not recirculate through LNs and perhaps had remained within this tissue, accepting that CD62L expression may have changed. To assess the cytokines produced by these populations, mice were again immunised and at 42dpi, cells from bLN and cLN were restimulated ex vivo. Following systemic infection with an attenuated Listeria monocytogenes expressing 2W1S, 2W1S‐specific Tem cells produce both IFN‐γ and IL‐2, whilst Tcm cells make only IL‐2 3. Consistent with their Tem phenotype based upon CD62L expression, the bLN was enriched for 2W1S‐specific T cells able to generate both IL‐2 and IFN‐γ whilst the cLN contained a 2W1S‐specific population that produced mostly IL‐2 only. Thus using a functional assessment of cytokine production, the 2W1S‐specific cells that had migrated to other LNs resembled a circulatory Tcm population, whilst in the draining bLN a more Tem‐like population appeared to be retained.

### Use of Kaede transgenic mice to assess CD4^+^ T‐cell recirculation

**Figure 3:**
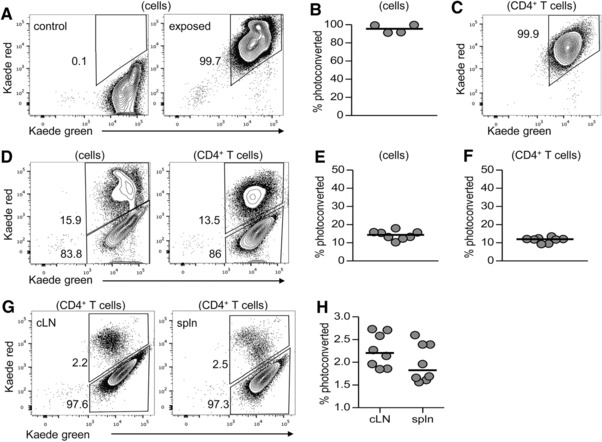
In situ labelling of cells in the bLN of Kaede transgenic mice. The left brachial LN (bLN) of Kaede transgenic mice was revealed under surgery and exposed to violet light (395 nm LED) for approximately 3 mins before the incision was sutured. (A–H) Mice were analysed (A–C) immediately or (D–H) 24 h after light exposure. (A) Expression of Kaede green versus Kaede red protein in cells isolated from the bLN immediately after violet light exposure or in non‐exposed controls was evaluated by flow cytometry. (B) Percentage of Kaede red^+^ lymphocytes in exposed bLN is shown. (C) Expression of Kaede green versus Kaede red protein in CD4^+^ T cells isolated from the exposed bLN. (D) Expression of Kaede green versus Kaede red protein in total cells (left) and CD4^+^ T cells (right) isolated from the exposed bLN. (E, F) The percentage of (E) Kaede red^+^ cells and (F) CD4^+^ T cells in exposed bLN. (G) Expression of Kaede green versus Kaede red in CD4^+^ T cells isolated from pool of contralateral LNs (cLN) or spleen (spln). (H) Percentage of Kaede red^+^ CD4^+^ T cells isolated from pool of cLN or spleen. Flow cytometry plots are representative of eight mice. Each symbol represents an individual mouse and data are pooled from two independent experiments; bars show medians.

Whilst phenotypic markers help discern the migratory potential of cells, it is now possible to temporally label cells at a given site using photoconversion, thanks to the development of photoconvertible Kaede transgenic mice 10. In these Kaede transgenic mice, violet light induces permanent structural changes to the Kaede chromophore resulting in altered excitation and emission wavelengths and a change from green to red fluorescence 10, 23. Thus cells may be labelled at a given site and their movement directly assessed. To validate our use of Kaede mice, the bLN was exposed by a small incision in the skin and then photoconverted. This resulted in Kaede red fluorescence being detected in the entire cellular compartment of the LN (Fig. 3A–C), demonstrating that all cells in the bLN had undergone photoconversion, consistent with the original studies 10. Analysis of the bLN 24 h post‐labelling revealed that only 10–15% of both total cells and CD4^+^ T cells remained Kaede red^+^ (Fig. 3D–F). Thus, the majority of the T cells present within the bLN 24 h post‐surgery were Kaede green^+^ and had entered the tissue in the 24‐h timeframe post photoconversion. Analysis of CD62L vs CD44 expression on CD4 T cells indicated that the vast majority of Kaede green^+^ CD4^+^ T cells were naive (CD62L^+^CD44^lo^) whilst the CD62L^−^CD44^hi^ Tem population was enriched amongst the Kaede red^+^ fraction (Supporting Information Fig. 1). Lymphocytes that had migrated from the bLN to other lymphoid tissues were identified as Kaede red^+^, and a distinct Kaede red^+^ population was evident in cLN and spleen, consistently accounting for approximately 2% of the total lymphocyte population (Fig. 3G and H). Again, the majority of these recirculating CD4^+^ T cells were of a CD62L^+^CD44^lo^ naïve phenotype. In the cLN Kaede red^+^ CD62L^−^CD44^hi^ cells were barely detectable, whilst this population was evident amongst the Kaede green^+^ population (Supporting Information Fig. 1). These data closely matched initial studies using the Kaede transgenic mice 10 and demonstrated robust labelling of cells in the bLN and the rapid recirculation of naïve CD4^+^ T cells through secondary lymphoid tissue.

**Figure 4:**
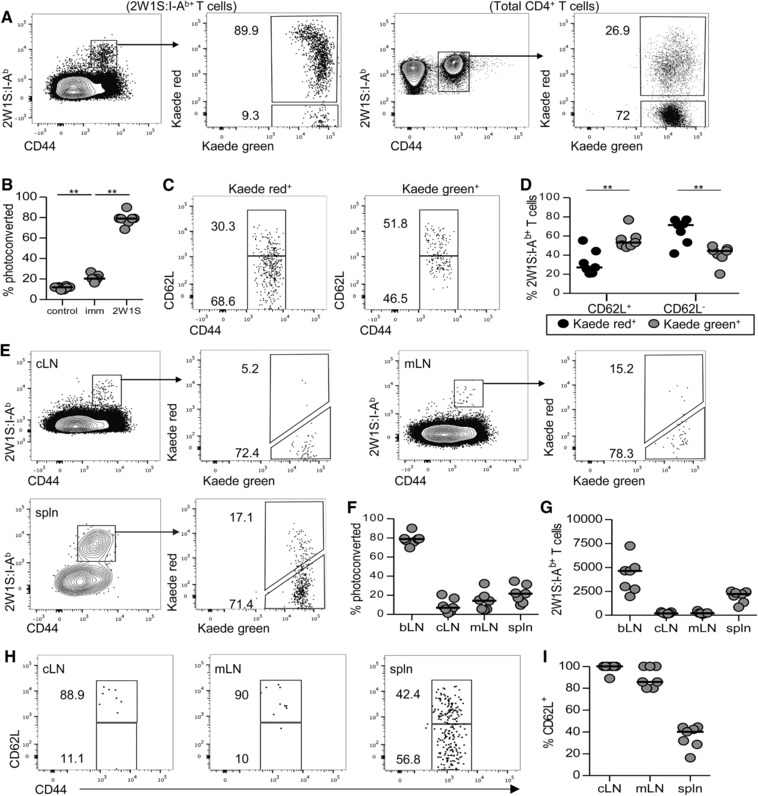
Rapid dissemination of 2W1S:I‐A^b+^ CD4^+^ T cells from the bLN. Kaede transgenic mice were immunised with 5 μg 2W1S peptide/alum in the left front paw pad and then the draining brachial LN (bLN) exposed to violet light at 8 dpi, with mice analysed 24 h later by flow cytometry. (A) Identification of Kaede green^+^ and Kaede red^+^ 2W1S‐specific and total CD4^+^ T cells in the bLN. (B) Percentage of photoconverted Kaede red^+^ CD4^+^ T cells isolated from control mice, immunised mice and those that are 2W1S‐specific in the bLN. (C) Expression of CD62L versus CD44 amongst Kaede green^+^ and Kaede red^+^ 2W1S:I‐A^b+^ CD4^+^ T cells in the bLN. (D) Percentage of Kaede green^+^ and Kaede red^+^ amongst CD62L^+^ and CD62L^−^ populations. (E) Identification of Kaede green^+^ and Kaede red^+^ 2W1S:I‐A^b+^ CD4^+^ T cells in a pool of contralateral LNs (cLN), mesenteric LNs (mLN) and spleen (spln). (F) Percentage of photoconverted Kaede red^+^ 2W1S:I‐A^b+^ CD4^+^ T cells in the different tissues. (G) Number of 2W1S:I‐A^b+^ CD4^+^ T cells in the different tissues. (H) Expression of CD62L versus CD44 amongst Kaede red^+^ 2W1S:I‐A^b+^ CD4^+^ T cells in the indicated tissues. (I) Percentage of CD62L^+^ Kaede red^+^ 2W1S:I‐A^b+^ CD4^+^ T cells in the indicated tissues. Flow cytometry plots are representative of 7 mice. (B D, F, G, I) Each symbol represents an individual mouse and data are pooled from two independent experiments; bars show medians. Mann–Whitney test: **p < 0.01, ***p < 0.001.

To track the migration of Ag‐specific CD4^+^ T cells, Kaede mice were immunised in the front paw pad with 2W1S peptide/alum, the bLN photoconverted at 8 dpi and tissues analysed 24 h later. Within the draining bLN, approximately 80% of the 2W1S‐specific CD4^+^ T cells population were Kaede red^+^ (Fig. 4A). When Kaede red expression in total CD4^+^ T cells was assessed, the proportion of Kaede red^+^ cells had significantly increased compared with bLNs of non‐immunised mice, indicating the impaired migration of all CD4^+^ T cells from the LN at this time (Fig. 4B). Kaede red^+^ 2W1S‐specific CD44^hi^ CD4^+^ T cells in the bLN mostly lacked expression of CD62L (median: 71.4%), whilst amongst Kaede green^+^ 2W1S‐specific CD44^hi^ CD4^+^ T cells, newly entered into the bLN within the last 24 h, a more equal mixture of CD62L^+^ and CD62L^−^ cells were detected (Fig. 4C and D). Kaede red^+^ 2W1S‐specific CD44^hi^ CD4^+^ T cells were detected in low numbers in all secondary lymphoid tissues analysed, constituting approximately 10–20% of the 2W1S‐specific population in cLN, mLN and spleen (Fig. 4E–G). Thus, a circulating 2W1S‐specific population of CD4^+^ T cells had been generated by 8 dpi. Notably, the vast majority of Kaede red^+^ 2W1S‐specific CD44^hi^ CD4^+^ T cells in the cLN and mLN were CD62L^+^ (>90%), consistent with the phenotype described for Tcm. Entry into the spleen is not dependent upon CD62L and here the Kaede red^+^ 2W1S‐specific CD44^hi^ CD4^+^ T cells were a mixture of CD62L^+^ and CD62L^−^ (Fig. 4H and I). Combined, these data demonstrate that by 8 dpi, activated Ag specific CD4^+^ T cells displaying the defining migratory characteristic of Tcm are already present.

### Establishment of a resident memory population within the draining LN

**Figure 5:**
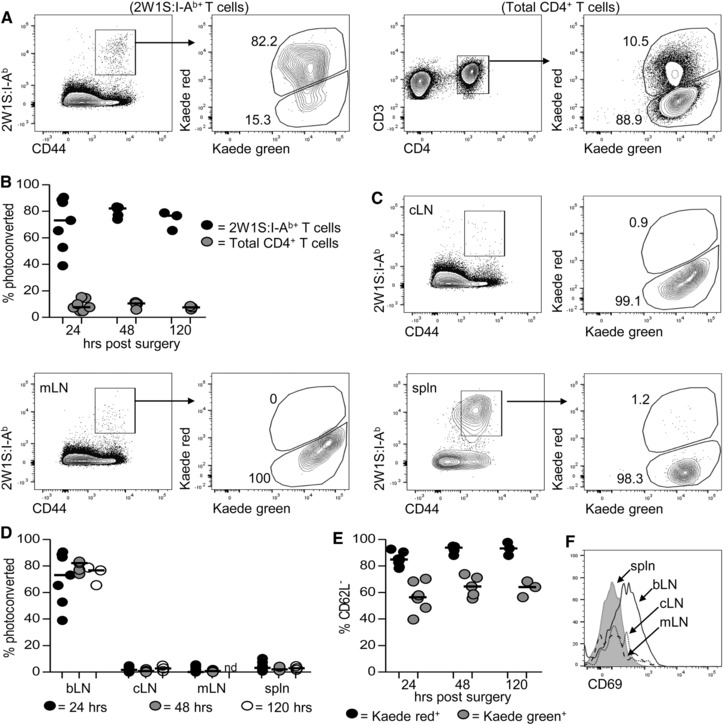
Memory 2W1S‐specific T cells persist within the draining bLN and do not recirculate. Kaede transgenic mice were immunised with 5 μg 2W1S peptide/alum in the left front paw pad and then the draining brachial LN (bLN) exposed to violet light at 42 dpi, with mice analysed 24, 48 and 120 h by flow cytometry. (A) Identification of Kaede green^+^ and Kaede red^+^ 2W1S‐specific and total CD4^+^ T cells in the bLN 48 h post exposure. (B) Percentage of Kaede red^+^ 2W1S‐specific and total CD4^+^ T cells in the bLN at 24, 48 and 120 h post light exposure. (C) Identification of Kaede green^+^ and Kaede red^+^ 2W1S:I‐A^b+^ CD4^+^ T cells isolated from a pool of contralateral LNs (cLN) mesenteric LNs (mLN) and spleen (spln) at 48 h post exposure. (D) Percentage of photoconverted Kaede red^+^ 2W1S:I‐A^b+^ CD4^+^ T cells in indicated tissues at 24,48 and 120 h post exposure. (E) Percentage of CD62L^−^ 2W1S:I‐A^b+^ CD4^+^ T cells expressing either Kaede red or Kaede green at 24, 48 and 120 h post exposure. (F) Expression of CD69 on 2W1S:I‐A^b+^ CD4^+^ T cells isolated from indicated tissues at 48 h post exposure. Flow cytometry plots are representative of at least five mice. Symbols represent individual mice and data are pooled from two (24 and 48 h) and one (120 h) independent experiment; bars show medians. Mann–Whitney test: **p < 0.01, ***p < 0.001, nd = not determined.

Analysis of Ag‐specific memory CD4^+^ T cells in the draining LN identified a population of cells that resembled Tem cells by surface markers and cytokine production. Given these cells lack expression of the molecules required to re‐enter LNs, it suggested these cells may have remained within the tissue. To directly test this, Kaede mice were immunised in the front paw pad with 2W1S peptide/alum and the bLN photoconverted at 42 dpi. The 2W1S‐specific populations in the draining bLN and other secondary lymphoid tissues were then assessed 24, 48 and 120 h later. Strikingly, the vast majority (median ∼75%) of 2W1S‐specific CD4^+^ T cells in the bLN remained Kaede red^+^ at all time points (Fig. 5A and B). These data demonstrated that this population had remained within the LN for at least 4 days consistent with a tissue resident population. Normal migration of non‐2W1S^+^ CD4^+^ T cells from the bLN was observed (Fig. 5B and C), indicating that the retention of the 2W1S‐specific memory population was peculiar to these Ag‐specific cells. Furthermore, when 2W1S‐specific populations were assessed in other secondary lymphoid tissues, Kaede red^+^ cells were not detected in neither cLN nor mLN and constituted only a very minor fraction of the population in the spleen (Fig. 5C). Thus, at 42 dpi, the CD62L^−^ 2W1S‐specific CD4^+^ T cells present in the draining LN do not leave this tissue and recirculate through other LNs in the 4 day time frame analysed (Fig. 5D and E). To test whether persistence of Ag at the injection site in the form of depots 24 might impact on the responding cells in the draining LN, mice were immunised in the paw pad with OVA‐2W1S and Rag × OTII cells transferred either immediately prior to, or 30 days after immunisation. Analysis of the OTII population (Supporting Information Fig. 2) provided a test of the presence of Ag depots able to stimulate naïve CD4^+^ T cells. OTII cells transferred 30 days post immunisation showed a modest increase in numbers compared with PBS controls, but substantially less than at 7 days post immunisation. These data indicate that whilst some Ag persists at 30 days post immunisation, much of the Ag injected is no longer present.

Whilst our analysis of the earlier time point demonstrated that a circulating 2W1S‐specific population resembling Tcm had been established after the first week of the response, this population may be below the sensitivity of detection with labelling at 42 dpi. Thus the majority of the 2W1S‐specific‐population in the bLN appears to have become resident. Earlier studies have linked CD69 expression to memory T‐cell tissue residency 25 and analysis of CD69 expression amongst 2W1S‐specific CD4^+^ T cells isolated from different tissues revealed substantially higher expression specifically in the bLN (Fig. 5F). To confirm that this putative tissue resident population was still detectable in the draining bLN at later time points, Kaede mice were immunised as before and the draining bLN labelled at 74 days post immunisation (Supporting Information Fig. 3). Again, the 2W1S‐specific population in the bLN at this time was predominantly Kaede red^+^ 48 h after photoconversion. The Kaede red^+^ population lacked CD62L expression and expressed higher levels of CD69 than 2W1S‐specific Kaede green^+^ cells in the bLN at this time (Supporting Information Fig. 3). These data again support the persistence of a distinct population of non‐migratory 2W1S‐specific CD4^+^ T cells for extended periods of time in the draining LN.

**Figure 6:**
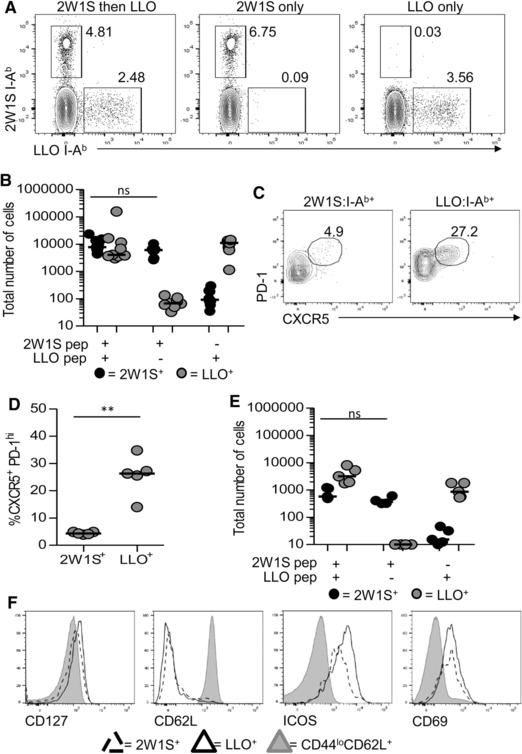
Ag‐specific memory populations residing in the draining LN are not out‐competed by subsequent responses. To assess the impact of a second T‐cell response on the LN resident 2W1S‐specific CD4^+^ T‐cell population, mice were first immunised with 5 μg 2W1S peptide/alum and then after approximately 42 days, immunised with 5 μg LLO peptide/alum, both in the left front paw pad. (A–F) Mice were then analysed at either (A–D) 8 or (E–F) 42 days post second immunisation. (A) Identification of 2W1S‐ and LLO‐specific CD4^+^ T‐cell populations in mice immunised with 2W1S then LLO peptide versus 2W1S‐only and LLO‐only controls (gated on CD3^+^CD4^+^CD44^hi^B220^−^CD11c^−^CD11b^−^ cells). (B) Numbers of 2W1S‐specific and LLO‐specific CD4^+^ T‐cell populations isolated from the bLN. (C) Expression of CXCR5 and PD‐1 by 2W1S‐specific and LLO‐specific CD4^+^ T‐cell populations (gated on Tetramer^+^CD3^+^CD4^+^CD44^hi^B220^−^CD11c^−^CD11b^−^ cells), isolated from the draining LN at 8 dpi with LLO peptide. (D) Percentage of CXCR5^+^PD‐1^hi^ T_FH_ cells. (E) Numbers of 2W1S‐specific and LLO‐specific CD4^+^ T‐cell populations isolated from the bLN. (F) Expression of CD127, CD62L, ICOS and CD69 amongst 2W1S‐specific, LLO‐specific and naïve (CD44^lo^CD62L^+^) CD4^+^ T cells in the bLN. Flow cytometry plots are all representative of at least five mice, values on plots are percentages. Symbols represent individual mice and data are pooled from two independent experiments; bars show medians. Mann–Whitney test: ns = non‐significant (*p* > 0.05), **p < 0.01.

These data indicate that following immunisation in the paw pad a population of Ag‐specific memory CD4^+^ T cells persists within the draining LN and form a resident memory population. These cells lack expression of the key molecules required for recirculation through other LNs and we found no evidence that these cells undertook such recirculation. These observations might be explained by cellular niches within the LN enabling retention of cells responsive to recently encountered Ags. Should such niches exist, the LN resident 2W1S‐specific population might be diminished upon exposure to a second Ag. To test this, C57BL/6 mice were first immunised with 2W1S peptide/alum as described previously, then immunised at 42 dpi with 5μg LLO peptide, again precipitated with alum. This second response stimulated an endogenous population of LLO‐specific CD4^+^ T cells previously described in C57BL/6 mice 3. Control mice that were immunised with only either 2W1S‐peptide or LLO peptide at the appropriate time were included for comparison. In mice immunised first with 2W1S, then LLO, two Ag‐specific populations of CD4^+^ T cells were evident (Fig. 6A). Importantly, the number of 2W1S‐specific CD4^+^ T cells in the bLN was not significantly different between mice immunised with both peptides, versus only the initial 2W1S peptide (Fig. 6B). These data indicate that initiation of a second T‐cell response in the draining LN did not diminish the putative LN resident memory population generated previously. Notably, analysis of T_FH_ cells amongst the Ag‐specific populations revealed that whilst a clear CXCR5^+^PD‐1^hi^ population was evident amongst the LLO‐specific population, the vast majority of the 2W1S‐specific memory population lacked markers associated with the T_FH_ phenotype (Fig. 6C), arguing against the notion that the persisting 2W1S‐specific memory cells resembled T_FH_ cells. To test whether development of a second LN memory population impacted on the 2W1S‐specific LN resident cells, mice were immunised first with 2W1S peptide/alum, then LLO peptide/alum (as described above), but assessed approximately 42 days after the second immunisation. Again, no loss of the 2W1S‐specific memory CD4^+^ T‐cell compartment was observed in mice that also contained memory LLO‐specific CD4^+^ T cells compared with mice immunised only with 2W1S/alum (Fig. 6E). Both the memory 2W1S‐specific and LLO‐specific CD4^+^ T cells in the bLN expressed enhanced levels of ICOS and CD69, comparable expression of CD127 and little CD62L compared with naïve CD4^+^ T cells (Fig. 6F). This phenotype is consistent with that recently described for a lymphoid tissue‐resident CD4^+^ T‐cell population detected in gut‐associated lymphoid tissue after prolonged oral Ag exposure 12. In summary our data reveal that following immunisation, peripheral LNs can generate a distinct population of memory CD4^+^ T cells that are retained within the draining LN and do not traffic to other lymphoid tissues. Thus further complexities exist in the migratory populations of memory CD4^+^ T‐cell populations generated during a response.

## Discussion

Direct *ex vivo* analysis of secondary lymphoid tissues reveals a mix of effector and memory CD4^+^ T cells populations based on a range of surface markers from which activation and migration capabilities can be proposed. Here we have attempted to better dissect these populations through limiting the site of the response to a specific peripheral LN and then combining assessment of endogenous CD4^+^ T cells detected with MHCII tetramers with site specific labelling of the responding cells using violet light in photoconvertible mice. We reasoned that this approach would clarify the extent to which CD4^+^ T cells were retained within the lymphoid tissue. Furthermore, it would test whether only a circulating memory population ultimately remained within LNs. Through these experiments, we identified an Ag‐specific memory CD4^+^ T‐cell population that persisted within the draining LN for several months and showed no evidence of leaving this tissue when migration was directly assessed. These cells showed phenotypic and functional similarities to Tem cells, producing both IFN‐γ and IL‐2 upon restimulation. These cells appeared quite distinct to T_FH_ cells. We conclude that following peripheral immunisation the draining LN retains an Ag‐specific Tem‐like population that appears to now be resident within the lymphoid tissue.

In these experiments, the use of Kaede transgenic mice enables site‐specific labelling, however, photoconversion only lasts for several days 10. Thus, whilst we have failed to detect evidence of migration over a period of days, it remains possible that the LN resident population shows migratory kinetics below the sensitivity of our assays. However, our data closely match an elegant recent study where long‐term *in vivo* cell tracking approach was developed using photoconvertible proteins tethered to histones to increase their stability 12. The authors here concluded that an effector/memory CD4^+^ T‐cell population exists as long‐term tissue resident cells within gut associated secondary lymphoid tissue such as Peyer's patches and LNs. Our data add to this initial description by demonstrating that a single immunisation in the periphery appears to also drive the formation of this lymphoid tissue resident population. The 2W1S‐specific population in the bLN described here closely resembles the cells identified by Ugur et al, sharing high expression of CD69, IL‐7Rα and ICOS in the absence of CD62L. Whist Ugur *et al*. and others 26 have described the persistence of T_FH_ populations as long term secondary lymphoid tissue resident cells, the memory 2W1S‐specific cells described here appear phenotypically distinct based on several surface markers. The role of Ag in this process may be important and Ugur et al. provided evidence that prolonged exposure to Ag supported the formation of this population. In the studies described here, alum was used as an adjuvant and given evidence that Ag‐depots may be formed with alum, more prolonged Ag exposure may exist in our experimental model 24. We do not think our data can be solely explained by Ag depots in the tissue since by 30 days post immunisation, naïve OTII cells were very poorly stimulated in the draining LN following transfer into immunised mice. Given that the putative LN resident population was evident within the draining LN for several months, our data from OTII responses indicates that the effects of Ag depots in the tissue have very likely expired.

A further striking observation of our data was the demonstration that CD44^hi^ Ag‐specific CD4^+^ T cells able to recirculate through LNs are formed within a few days of the response. Whilst the recall responses of these cells were not assessed, these cells clearly possessed a defining feature of Tcm, that is, the ability to recirculate through secondary lymphoid tissue. These data are consistent with the rapid generation of memory population during the response rather than cells becoming a memory population after a certain timespan served as effector cell.

What is the purpose of retaining such a population within the LN? Perhaps a resident memory T‐cell population can rapidly produce effector cytokines within the tissue to potentiate secondary responses, augmenting the response from innate populations such as innate lymphoid cells 27. Such responses may have evolved to provide further protection to the host should the same insult be repeated. Identifying where these memory cells reside within the tissue and how they differ from other memory T‐cell populations may also shed light on the contribution these cells make to immune responses. Given the predominance of peripheral immunisation in current vaccination approaches, our data reveal a further CD4^+^ T‐cell population to be considered amongst the responding cells.

## Materials and methods

### Mice

All mice used were bred and maintained in accordance with Home Office guidelines at the University of Birmingham, Biomedical Services Unit. Mice used were: C57BL/6, Kaede transgenic 10 and CD45.1 Rag × OTII which are on a C57BL/6 background.

### Immunisation

Mice were immunised with peptide precipitated with alum (using 9% aluminium sulphate) or OVA conjugated to 2W1S, in a volume of 10 μL into the front paw pad as indicated.

### Surgical procedures

Mice were anesthetised and a small incision made in the skin directly above the bLN on the left side of the mouse. The bLN was then exposed to violet light (395 nm UV LED) for approximately 3 min. The incision was then sutured and mice analysed at specified times post‐surgery. Before surgery, the mice received s.c. Buprenorphine (Vetergesic, Reckitt Benckiser Healthcare) at 0.15 mL per 100 g as analgesic.

### Cell preparation and ex vivo stimulation

All LNs were cleaned of fat and then pulled apart using fine forceps and the resulting cell suspension passed through 70 μm nylon mesh with fragments crushed with a syringe plunger. Spleens were crushed through 70 μm nylon mesh with a syringe plunger and red blood cells in the resulting cell suspension lysed using Gey's solution. To assess cytokine secretion, cells were cultured in the presence of 50 ng/mL PMA (Sigma) and 750 ng/mL ionomycin (Sigma) for 1 h, after which Brefeldin A (Sigma) to a final concentration of 10ug/mL was added and cells cultured for a further 3 h.

### Flow cytometry

Where indicated, cells were first stained with MHCII tetramers (2W1S:I‐A^b^ and LLO:I‐A^b^) diluted 1:100 in staining buffer (PBS/2% FCS/2.5 mM EDTA) for approximately 1 h at RT. Cell surface staining was done at 4°C for 30 min, except for CXCR5 (clone 2G8, BD Biosciences) which was stained for 1 h at RT. 2W1S:I‐A^b+^ CD4^+^ T cells in the spleen were enriched as described previously 14, 28, using anti‐PE or ‐APC MicroBeads (Miltenyi Biotech) and MACS enrichment. 2W1S:I‐A^b+^ and LLO:I‐A^b+^ CD4^+^ T cells were identified amongst cells that were B220^−^ (clone RA3‐6B2, Ebioscience), CD11b^−^ (clone M1170, Ebioscience), CD11c^−^ (clone N418, Ebioscience), CD3^+^ (clone 17A2, Ebioscience), CD4^+^ clone RM4‐5, Ebioscience and Biolegend) and CD44^hi^ (clone 1M7, Ebioscience). Further Abs used were: anti‐mouse CD62L (clone MEL‐14, Ebioscience), CD69 (clone HI.2F3, Ebioscience and Biolegend), ICOS (clone 15F9, Ebioscience and Biolegend), IL‐2 (clone JES6‐5H4, BD Biosciences), IFN‐γ (clone 4S.B3, eBioscience), PD‐1 (clone RMPI‐30, Biolegend). Intracellular staining for cytokines was done as described previously 29. Samples were collected using a Fortessa X20 (BD Biosciences) and analysed using FlowJo software (Tree Star). Spherotech Accucount blank particles were added to each sample to calculate cell frequency. To identify Kaede red^+^ and Kaede green^+^ 2W1S‐specific CD4^+^ T cells, gates were set based on total CD4^+^ T cells, where clear populations were evident. Mice in which clear segregation of Kaede red^+^ and Kaede green^+^ populations was not evident amongst the total CD4^+^ T cells, occasionally observed due to technical errors when labelling the LN during surgery, were excluded from subsequent analyses.

### Statistics

Data were analysed using GraphPad Prism (version 6.0e). Non‐parametric two‐tailed Mann–Whitney test was used to determine significance which was set at *p* ≤ 0.05. Median values were calculated and used in all analyses.

## Conflict of interest

The authors declare no financial or commercial conflict of interest.

**Abbreviations**
b: brachial
c: contralateral
DC: dendritic cell
dpi: days post immunisation
LN: lymph node
m: mesenteric
Tcm: T central memory
Tem: T effector memory
Trm: T resident memory
T_FH_: T follicular helper

## Supporting information


**Supporting Information Figure 1**. Migration of CD4+ T‐cell subsets 24 h following photoconversion of the brachial LN. The left brachial LN (bLN) of Kaede mice was exposed to violet light for 3 min. 24 h later, the bLN, a pool of contralateral LNs (cLN) and the spleen were analysed. (A) Expression of CD62L versus CD44 amongstKaede red and Kaede green CD4+ T cells from the bLN. (B) Percentage of populations identified on basis of CD62L versus CD44 expression amongst Kaede red and Kaede green CD4+ T‐cell populations in the bLN. (C) Expression of CD62L versus CD44 amongst Kaede red and Kaede green CD4+ T cells in the cLN. (D) Percentage of populations identified on basis of CD62L versus CD44 expression amongst Kaede red and Kaede green CD4+ T‐cell populations in the cLN. (E) Expression of CD62L versus CD44 amongst Kaede red and Kaede green CD4+ T cells in the spleen. (F) Percentage of populations identified on basis of CD62L versus CD44 expression amongst Kaede red and Kaede green CD4+ T‐cell populations in the spleen. (A, C, E) Plots are representative of 8 mice from 2 independent experiments. Values on plots are percentages. (B, D, F) Graphs showed pooled data from 2 independent experiments. Symbols represent individual mice, bars show median. Mann Whitney Test: *p ≤ 0.05, **p ≤ 0.01, ***p ≤ 0.001, ns= non‐significant.
**Supporting Information Figure 2**. Immunisation with OVA‐2W1S/alum in the paw pad results in minimal antigen depots capable of supporting naive T‐cell expansion 30 days later. C57BL/6 WT mice were immunised in the left paw pad with 5ƒÊg OVA‐2W1S precipitated with alum. Miceadditionally received either PBS or 50,000 CD45.1+ OTII cells from Rag x OTII mice i.v. 24 h prior to, or30 days after the OVA‐2W1S immunisation. Numbers of activated OTII cells (CD45.1+CD3+CD4+CD44hi cells) were analysed at 7 days after the initial immunisation or 7 days after transfer of OTII cells at 30 days post immunisation. (A) Schematic of experimental design. (B) Representative flow cytometry plots showing OTII and 2W1S‐specific CD4+ T‐cell populations. (C) Numbers of OTII cells recovered from mice immunised with PBS or 5ƒÊg OVA‐2W1S at D0 or D30 time points. Graph shows pooled data from 2 independent experiments at D30 and 1 experiment at D0. Symbols represent individual mice, bars show median.
**Supporting Information Figure 3**. Non‐migratory 2W1S‐specific CD4+ T cells are retained in the draining LN beyond 70 days post immunisation. Kaede mice were immunised in the left paw pad with 5μg 2W1S peptide precipitated with alum. At 74 days post immunisation, the left bLN was exposed under surgery and photoconverted. Mice were analysed 48 h later and the draining bLN and a pool of contralateral LNs (cLN; containing axillary, brachial, and inguinal) analysed. (A) Representative expression of Kaede red and Kaede green amongst 2W1S‐specific CD4+ T cells in draining bLN and cLN, as well as expression of CD62L and CD69 by these populations. (B) Percentage of photoconverted (Kaede red+) 2W1S‐specific CD4+ T cells from the draining bLN and cLN. (C, D) Percentage of (C) CD69+CD62L‐ and (D) CD69‐CD62L+ amongst Kaede red and green 2W1S‐specific CD4+ T cells from the draining bLN. (E) Numbers of 2W1S‐specific CD4+ T cells recovered from the draining bLN and cLN. Symbols represent individual mice, bars show median. Mann Whitney Test: *p ≤ 0.05.

Peer review correspondence
