# Supplementary material for: Retention of Ag‐specific memory CD4+ T cells in the draining lymph node indicates lymphoid tissue resident memory populations
Source: Eur J Immunol. 2017 Apr 11;47(5):860–71. doi: 10.1002/eji.201646681 (PMC5435927; doi:10.1002/eji.201646681)
Supplement: Supplementary file 1 — Supporting Information Figure 1. Migration of CD4+ T‐cell subsets 24 h following photoconversion of the brachial LN. The left brachial LN (bLN) of Kaede mice was exposed to violet light for 3 min. 24 h later, the bLN, a pool of contralateral LNs (cLN) and the spleen were analysed. (A) Expression of CD62L versus CD44 amongstKaede red and Kaede green CD4+ T cells from the bLN. (B) Percentage of populations identified on basis of CD62L versus CD44 expression amongst Kaede red and Kaede green CD4+ T‐cell populations in the bLN. (C) Expression of CD62L versus CD44 amongst Kaede red and Kaede green CD4+ T cells in the cLN. (D) Percentage of populations identified on basis of CD62L versus CD44 expression amongst Kaede red and Kaede green CD4+ T‐cell populations in the cLN. (E) Expression of CD62L versus CD44 amongst Kaede red and Kaede green CD4+ T cells in the spleen. (F) Percentage of populations identified on basis of CD62L versus CD44 expression amongst Kaede red and Kaede green CD4+ T‐cell populations in the spleen. (A, C, E) Plots are representative of 8 mice from 2 independent experiments. Values on plots are percentages. (B, D, F) Graphs showed pooled data from 2 independent experiments. Symbols represent individual mice, bars show median. Mann Whitney Test: *p ≤ 0.05, **p ≤ 0.01, ***p ≤ 0.001, ns= non‐significant. Supporting Information Figure 2. Immunisation with OVA‐2W1S/alum in the paw pad results in minimal antigen depots capable of supporting naive T‐cell expansion 30 days later. C57BL/6 WT mice were immunised in the left paw pad with 5ƒÊg OVA‐2W1S precipitated with alum. Miceadditionally received either PBS or 50,000 CD45.1+ OTII cells from Rag x OTII mice i.v. 24 h prior to, or30 days after the OVA‐2W1S immunisation. Numbers of activated OTII cells (CD45.1+CD3+CD4+CD44hi cells) were analysed at 7 days after the initial immunisation or 7 days after transfer of OTII cells at 30 days post immunisation. (A) Schematic of experimental design. (B) Representative f [file EJI-47-860-s001.pdf]

European Journal of Immunology

Supporting Information
for
DOI 10.1002/eji.201646681

Clare L. Marriott, Emma E Dutton, Michio Tomura and David R. Withers

Retention of Ag-specific memory $CD4^{+}$ T cells in the draining lymph node indicates lymphoid tissue resident memory populations

© 2017 The Authors. *European Journal of Immunology* published by WILEY-VCH Verlag GmbH & Co. KGaA, Weinheim.

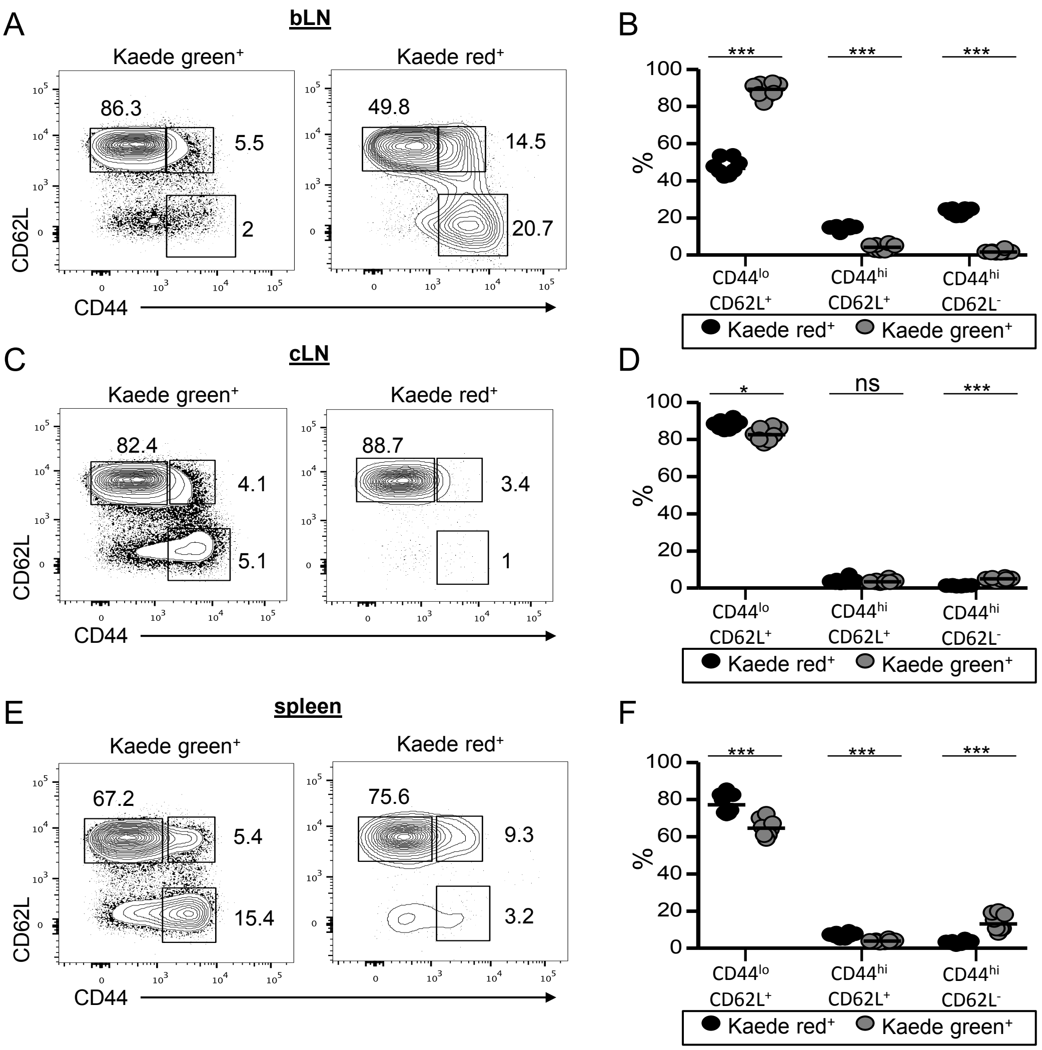

Supporting Information Figure 1: Migration of CD4$^+$ T cell subsets 24 hours following photoconversion of the brachial LN.

The left brachial LN (bLN) of Kaede mice was exposed to violet light for 3 minutes. 24 hours later, the bLN, a pool of contralateral LNs (cLN) and the spleen were analysed. (A) Expression of CD62L versus CD44 amongst Kaede red and Kaede green CD4$^+$ T cells from the bLN. (B) Percentage of populations identified on basis of CD62L versus CD44 expression amongst Kaede red and Kaede green CD4$^+$ T cell populations in the bLN. (C) Expression of CD62L versus CD44 amongst Kaede red and Kaede green CD4$^+$ T cells in the cLN. (D) Percentage of populations identified on basis of CD62L versus CD44 expression amongst Kaede red and Kaede green CD4$^+$ T cell populations in the cLN. (E) Expression of CD62L versus CD44 amongst Kaede red and Kaede green CD4$^+$ T cells in the spleen. (F) Percentage of populations identified on basis of CD62L versus CD44 expression amongst Kaede red and Kaede green CD4$^+$ T cell populations in the spleen. (A, C, E) Plots are representative of 8 mice from 2 independent experiments. Values on plots are percentages. (B, D, F) Graphs showed pooled data from 2 independent experiments. Symbols represent individual mice, bars show median. Mann Whitney Test: *p≤0.05, **p≤0.01, ***p≤0.001, ns= non-significant.

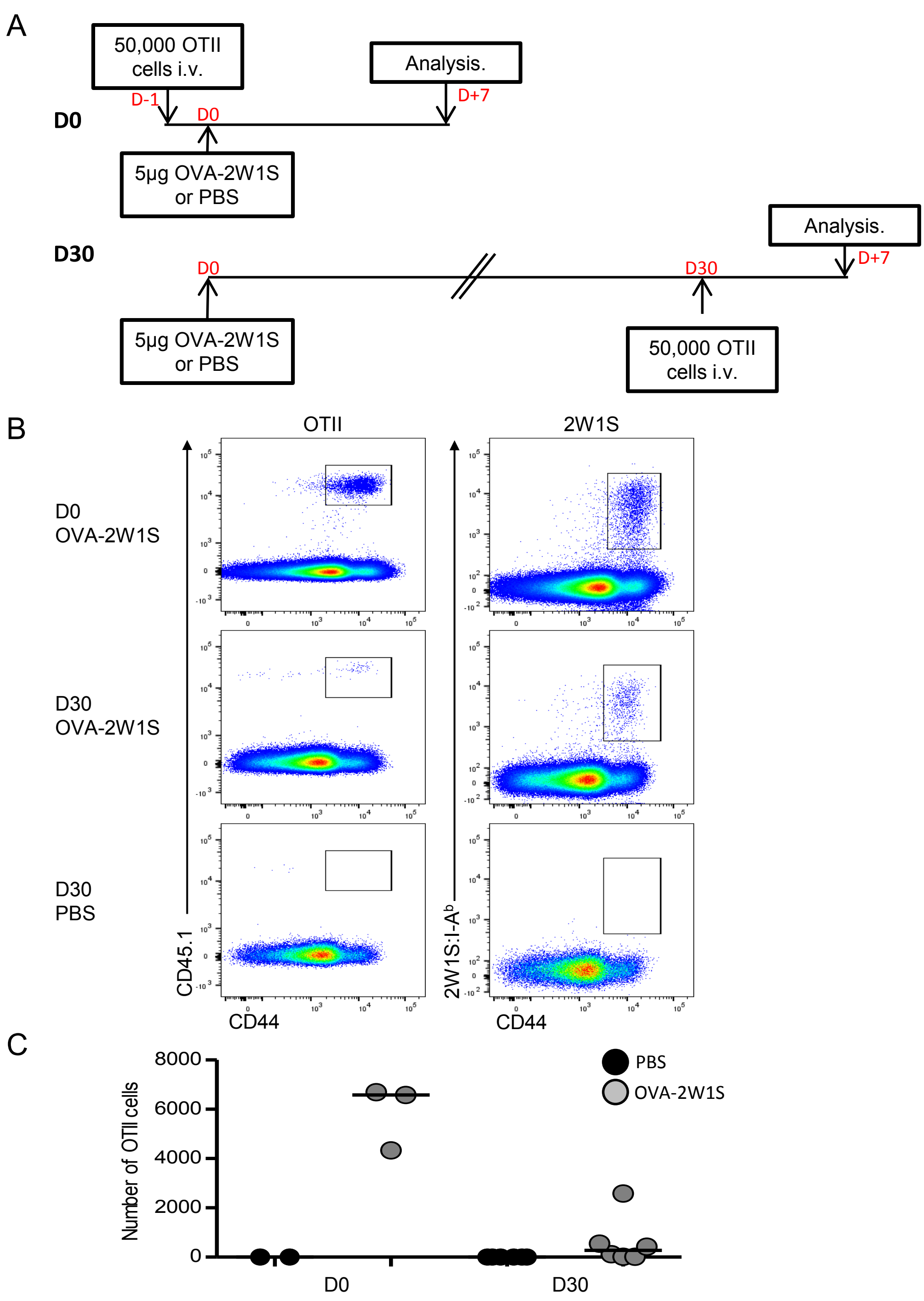

Supporting Information Figure 2: Immunisation with OVA-2W1S/alum in the paw pad results in minimal antigen depots capable of supporting naïve T cell expansion 30 days later.

C57BL/6 WT mice were immunised in the left paw pad with 5µg OVA-2W1S precipitated with alum. Mice additionally received either PBS or 50,000 CD45.1^{+} OTII cells from Rag x OTII mice i.v. 24 hours prior to, or 30 days after the OVA-2W1S immunisation. Numbers of activated OTII cells (CD45.1^{+}CD3^{+}CD4^{+}CD44^{hi} cells) were analysed at 7 days after the initial immunisation or 7 days after transfer of OTII cells at 30 days post immunisation. (A) Schematic of experimental design. (B) Representative flow cytometry plots showing OTII and 2W1S-specific CD4^{+} T cell populations. (C) Numbers of OTII cells recovered from mice immunised with PBS or 5µg OVA-2W1S at D0 or D30 time points. Graph shows pooled data from 2 independent experiments at D30 and 1 experiment at D0. Symbols represent individual mice, bars show median.

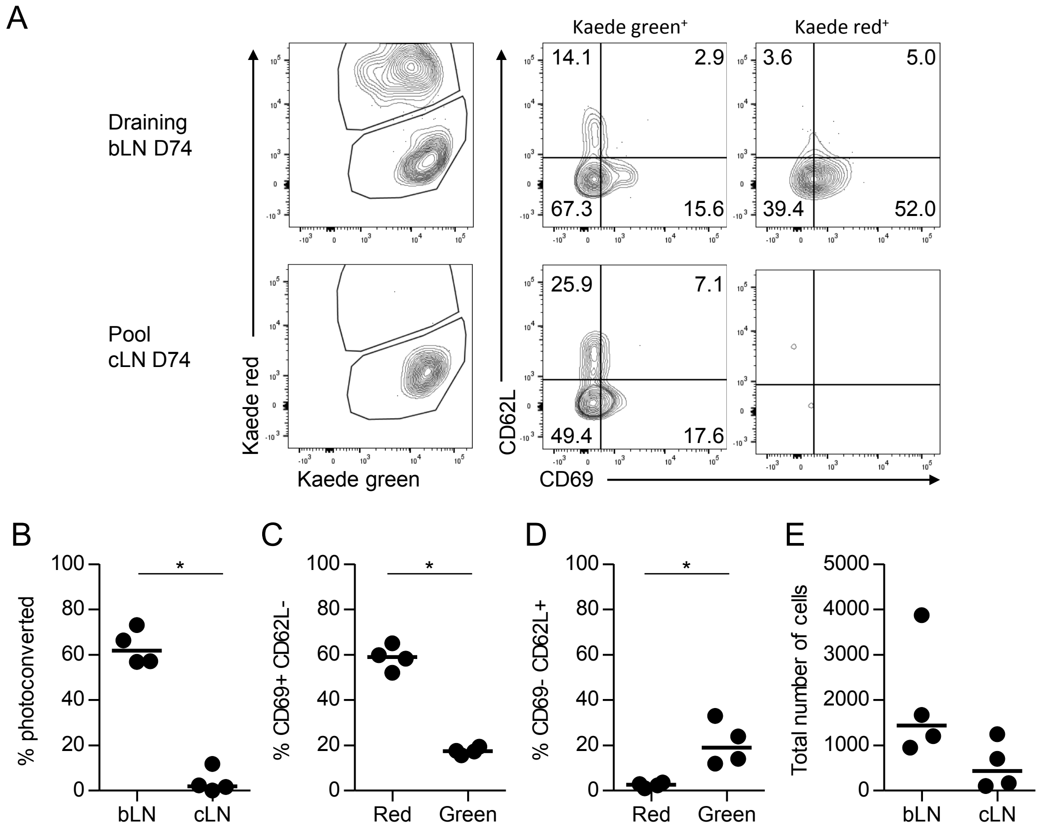

Supporting Information Figure 3: Non-migratory 2W1S-specific CD4+ T cells are retained in the draining LN beyond 70 days post immunisation.

Kaede mice were immunised in the left paw pad with 5µg 2W1S peptide precipitated with alum. At 74 days post immunisation, the left bLN was exposed under surgery and photoconverted. Mice were analysed 48 hours later and the draining bLN and a pool of contralateral LNs (cLN; containing axillary, brachial, and inguinal) analysed. (A) Representative expression of Kaede red and Kaede green amongst 2W1S-specific $CD4^+$ T cells in draining bLN and cLN, as well as expression of CD62L and CD69 by these populations. (B) Percentage of photoconverted (Kaede red^+) 2W1S-specific $CD4^+$ T cells from the draining bLN and cLN. (C,D) Percentage of (C) $CD69^+CD62L^-$ and (D) $CD69^-CD62L^+$ amongst Kaede red and green 2W1S-specific $CD4^+$ T cells from the draining bLN. (E) Numbers of 2W1S-specific $CD4^+$ T cells recovered from the draining bLN and cLN. Symbols represent individual mice, bars show median. Mann Whitney Test: *p≤0.05
