# Supplementary material for: Retention of Ag‐specific memory CD4+ T cells in the draining lymph node indicates lymphoid tissue resident memory populations
Source: Eur J Immunol. 2017 Apr 11;47(5):860–71. doi: 10.1002/eji.201646681 (PMC5435927; doi:10.1002/eji.201646681)
Supplement: Supplementary file 2 — Peer review correspondence [file EJI-47-860-s002.pdf]

Peer review correspondence

European Journal of Immunology

Retention of Ag-specific memory $CD4^+$ T cells in the draining lymph node indicates lymphoid tissue resident memory populations

Clare L. Marriott, Emma E. Dutton, Michio Tomura and David R. Withers

Corresponding author: David R. Withers, Institute of Immunology & Immunotherapy (III), College of Medical and Dental Sciences, University of Birmingham, Birmingham, United Kingdom

---

Review Timeline:	Submission date:	17 August 2016
	First editorial decision:	13 September 2016
	Revision received:	3 February 2017
	Accepted:	8 March 2017

Handling Executive Committee member: Prof. Shimon Sakaguchi

Please note that the correspondence below does not include the standard editorial instructions regarding preparation and submission of revised manuscripts, only the scientific revisions requested and addressed.

First Editorial Decision – 13 September 2016

Dear Dr. Withers,

Manuscript ID eji.201646681 entitled "Retention of antigen-specific memory CD4+ T cells in the draining lymph node indicates lymphoid tissue resident memory populations" which you submitted to the European Journal of Immunology has been reviewed. The comments of the referees are included at the bottom of this letter.

A revised version of your manuscript that takes into account the comments of the referees will be reconsidered for publication. You will see that Referee 1 feels that a major caveat needs to be addressed, while Referee 2 sees that your work is confirmatory of reference [12] in your submission. This referee feels that although your study is well-performed, it presents an incremental advance over previously published work; we see that you already mention Ugur et al. in your Discussion but would be grateful if you could more explicitly state the advance which your data present over that of Ugur et al.

Should you disagree with any of the referees' concerns, you should address this in your point-by-point response and provide solid scientific reasons for why you will not make the requested changes.

You should also pay close attention to the editorial comments included below. *In particular, please edit your figure legends to follow Journal standards as outlined in the editorial comments. Failure to do this will result in delays in the re-review process.*

Please note that submitting a revision of your manuscript does not guarantee eventual acceptance, and that your revision will be re-reviewed by the referees before a decision is rendered.

If the revision of the paper is expected to take more than three months, please inform the editorial office. Revisions taking longer than six months may be assessed by new referees to ensure the relevance and timeliness of the data.

Once again, thank you for submitting your manuscript to European Journal of Immunology and we look forward to receiving your revision.

Yours sincerely,
Karen Chu

On behalf of Prof. Shimon Sakaguchi

Dr. Karen Chu
Editorial Office
European Journal of Immunology
e-mail: ejied@wiley.com
www.eji-journal.eu

****

Reviewer: 1

Comments to the Author
Marriott et al. have used peptide/alum immunization in combination with photo-convertible KAEDE mice to investigate antigen-specific CD4+ T cell responses generated in skin-draining LN. They observe that CD4+ T cells with an effector memory phenotype are retained in LN draining the site of immunization for several months without appreciable migration over a period of several days. Based on these findings, the

European Journal of Immunology

authors conclude that peripheral immunization generates a type of lymphoid tissue resident memory CD4+ T cell population.

While their study is generally of interest, there remains a major caveat. The authors need to rule out that their findings are not simply explained by the persistence of antigen in the LN, possibly due to continuous release from the peripheral antigen/alum depot. I have more specific comments and questions relating to this concern below.

SPECIFIC COMMENTS:
Given the phenotype and cytokine profile of SW1S-specific CD4+ T cells in bLN 42 p.i. it appears very likely that antigen is still present in the LN, possibly being released from the injection site over several weeks. If that were true, retention and the non-migratory phenotype of the cells would be explained by continuous TCR stimulation on effector cells, as opposed to an intrinsically distinct migration phenotype of memory CD4+ T cells. The expression of CD69 (Figure 6F) further supports this possibility. Is there continuous proliferation of SW1S-specific CD4+ T cells compared with other CD44hi CD4+ T cells in this and other sites at late time points p.i.? Can resting or naïve SW1S-specific cells be transferred and tested for specific activation in bLN at late time points p.i. to probe for persisting antigen?

Related to the previous comment, there is a considerable attrition of SW1S-specific CD4+ T cells between d50 and d84 p.i. from approximately 7,000 to 700 cells, respectively (compare Figure 6B and 6E). This further implies that cells at 6 weeks p.i. have not yet formed a stable memory population and that slowly decreasing antigen levels are the cause of their gradual disappearance. Does photo-conversion at these late time points give similar results as in Figure 5?

It remains unclear why the authors use the term “memory” for cells they isolate 9d p.i. (e.g. relating to Figure 4).

Are the SW1S-specific CD4+ T cells in Figure 1A predominantly PD-1-negative, as stated or are their PD-1-intermediate? What is the PD-1 expression level on other CD4+ T cells in the same location?

Reviewer: 2

Comments to the Author
In this study, Withers and coworkers use a front footpad immunization and brachial LN photoconversion approach to study recirculation versus residence of responding LN T cells. They find that a large fraction of antigen specific memory CD4+ T cells are resident in the draining LN. This includes many cells that do

European Journal of Immunology

not have a Tfh phenotype. These data are consistent with other studies that have demonstrated the presence of LN resident effector memory cells (e.g. Ugur et al., Nat. Comm. 2014). By using a different immunization approach and tracking the phenotype of the antigen specific cells, these data represent an incremental advance over the previous work. The work is considered well performed though the findings do not provide substantial new insight regarding the mechanism of residence or its significance.

First revision – authors' response – 3 February 2017

Response to Reviewers comments

We thank the reviewers for their supportive comments. We have responded to each of these below, with the original reviewer comment displayed first. We have amended our original manuscript with changes to the text now shown in red. Two new Supporting Figures have been included to directly assess the concerns of Reviewer 1. The experiments performed where as requested by the reviewer. We have completely re-written the discussion section to better describe what we think our data means and how it adds to our current understanding of memory T cell populations. We consider our manuscript to be further improved by the additional experimental data.

Reviewer 1:

QUESTION 1: Given the phenotype and cytokine profile of SW1S-specific CD4+ T cells in bLN 42 p.i. it appears very likely that antigen is still present in the LN, possibly being released from the injection site over several weeks. If that were true, retention and the non-migratory phenotype of the cells would be explained by continuous TCR stimulation on effector cells, as opposed to an intrinsically distinct migration phenotype of memory CD4+ T cells. The expression of CD69 (Figure 6F) further supports this possibility. Is there continuous proliferation of SW1S-specific CD4+ T cells compared with other CD44hi CD4+ T cells in this and other sites at late time points p.i.? Can resting or naïve SW1S-specific cells be transferred and tested for specific activation in bLN at late time points p.i. to probe for persisting antigen?

RESPONSE:
We agree with the reviewer that the role of antigen in our model is a potential caveat that should be addressed. Without transferring cells from one mouse to another, it is almost impossible to rule out a role for antigen in any in vivo system. In our model the use of endogenous cells makes transfers between mice technically very challenging. Furthermore, what this data means is highly questionable when putative tissue resident populations are being assessed – the transfer would require the cells to be ripped out of their normal environment in the tissue.

European Journal of Immunology

The experiment proposed by the reviewer is a good one and we have a performed a slightly amended version that takes into account the challenges of transferring endogenous CD4 T cell populations and the ease with which TCR transgenic T cells can be transferred. Thus, mice received 50,000 Rag x OTII cells (CD45.1+) i.v. and then 24hrs later mice were immunised in the paw pad as previously with alum precipitated OVA-2W1S. This approach enables both the OTII and 2W1S response to be assessed. The number of OTII cells (CD45.1+ CD44hi CD4+ T cells) and 2W1S-specific cells was then analysed at day 7 post immunisation. Other mice, in which the paw pad was immunised as before, received 50,000 Rag x OTII cells (CD45.1+) i.v. at 30 days post immunisation and then were analysed 7 days later. Thus a naïve pool of antigen-specific CD4 T cells were assessed for their ability to become activated and expand early and later in the response, providing a test for the presence of antigen in the amounts needed to stimulate naïve CD4 T cells.

This data is now included as Supporting Figure 2. We observed very limited expansion and activation of OTII cells transferred 30 days after immunisation. This was in contrast to the robust expansion observed when the OTII cells were present at the time of immunisation. Thus by 30 days post immunisation the levels of antigen in the tissue have dramatically reduced, arguing against depots of antigen supporting continuous T cell activation. We conclude that this weak activation of antigen specific naïve CD4 T cells strongly argues against our data on the 2W1S response merely reflecting continuous release of antigen from the injection site over time. This new data is described on Page 10 and we have further recognised this in our entirely revised discussion.

QUESTION 2: Related to the previous comment, there is a considerable attrition of SW1S-specific CD4+ T cells between d50 and d84 p.i. from approximately 7,000 to 700 cells, respectively (compare Figure 6B and 6E). This further implies that cells at 6 weeks p.i. have not yet formed a stable memory population and that slowly decreasing antigen levels are the cause of their gradual disappearance. Does photo-conversion at these late time points give similar results as in Figure 5?

RESPONSE:
We have performed the photo-conversion experiment proposed by the reviewer at the later time point of 74 days post immunisation with OVA-2W1S, with analysis 48hrs after labelling. These data are shown in Supporting Figure 3 and show that the majority of 2W1S-specific CD4 T cells at this time point persist within the tissue as observed at 42 days post immunisation. Thus this draining LN population of CD44hi 2W1S-specific T cells likely reside in this tissue and do not circulate through lymph nodes with the kinetics of naïve T cells or cells with the phenotype of central memory T cells. We consider these data to further support out initial conclusion that the draining brachial LN retains a population of effector memory-like cells for many months after immunisation. The text on Page 10 has been amended to describe this data.

QUESTION 3: It remains unclear why the authors use the term “memory” for cells they isolate 9d p.i. (e.g. relating to Figure 4).

RESPONSE:
We take the reviewers point here that we have not proved that the cells are capable of a rapid secondary response upon re-encounter of antigen and thus the term ‘memory’ may be presumptive. We used the term ‘memory since our data demonstrates that these are CD4 T cells that have clearly encountered antigen (and thus are no longer naïve) and are able to recirculate through secondary lymphoid tissue. We directly test this given our ability to label them in one LN and detect them in contralateral LNs. Thus, these CD44hi CD4 T cells are behaving functionally in the manner ascribed to central memory T cells, that of recirculation through secondary lymphoid tissue.

We would make the point that other studies have concluded that memory cells are rapidly formed during a response and models of CD4 T cell responses in vivo do not support a model where memory populations are only formed after a certain time. The functional measure of recirculation that our studies of Kaede mice enable, provides supporting data that these are central memory cells. Since we have not been able to track this specific population through to restimulation studies, we have amended our description of these data to simply state that the cells display functional capabilities consistent with central memory T cells.

QUESTION 4: Are the SW1S-specific CD4+ T cells in Figure 1A predominantly PD-1-negative, as stated or are their PD-1-intermediate? What is the PD-1 expression level on other CD4+ T cells in the same location?

RESPONSE:
We thank the reviewer for pointing out this error in our description of the data. The level of PD-1 expression on 2W1S-specific CD4+ T cells in Figure 1A (day 8 post immunisation) was higher than other CD4+ T cells in the same location. We have amended this in the original text to intermediate rather than negative. Other PD-1 staining is also evident in Figure 6C, where the intermediate levels seen on recently responding LLO-specific cells (at a time point comparable to that in Figure 1A) contrasts with the lack of PD-1 expression seen on the memory 2W1S+ cells (‘true’ PD-1-negative cells).

European Journal of Immunology

Reviewer: 2

The reviewer states that:

These data are consistent with other studies that have demonstrated the presence of LN resident effector memory cells (e.g. Ugur et al., Nat. Comm. 2014). By using a different immunization approach and tracking the phenotype of the antigen specific cells, these data represent an incremental advance over the previous work. The work is considered well performed though the findings do not provide substantial new insight regarding the mechanism of residence or its significance.

RESPONSE:
The work of Ugur et al. provided the first direct evidence of tissue resident memory CD4 T cells in the secondary lymphoid tissue associated with the intestine. Our study demonstrates that this lymphoid tissue resident memory population is also apparent in the peripheral draining lymph nodes. This is of clear relevance given that a large number of vaccines stimulate peripheral responses and our data has substantial implications for the nature of the memory responses generated. A key novel aspect of our work is the tracking of an endogenous antigen specific population of CD4+ T cells. Such responses are far more physiological than the use of TCR transgenics (as for example in Ugur et al. 2014) and much more likely to correctly inform responses in human patients. Thus whilst our data is consistent with that of Ugur et al., we would argue that our data significantly builds on this study, both in the power of the analysis and in expanding our understanding of the locations that lymphoid resident populations can persist. We think our data is of real relevance to understanding the memory responses generated through peripheral vaccination, a topic of relevance to the broad immunological community.

We would agree with the reviewer that our study does not address the mechanisms that control tissue residency. To our understanding, these are poorly understood for all populations of tissue resident immune cells and whilst this is an important question, we would politely suggest this is beyond the scope of our current study.

Peer review correspondence

European Journal of Immunology

Second Editorial Decision – 20 February 2017

Dear Dr. Withers,

It is a pleasure to provisionally accept your manuscript entitled "Retention of antigen-specific memory CD4+ T cells in the draining lymph node indicates lymphoid tissue resident memory populations" for publication in the European Journal of Immunology. For final acceptance, please follow the instructions below and return the requested items as soon as possible as we cannot process your manuscript further until all items listed below are dealt with.

Please note that EJI articles are now published online a few days after final acceptance (see Accepted Articles: http://onlinelibrary.wiley.com/journal/10.1002/(ISSN)1521-4141/accepted). The files used for the Accepted Articles are the final files and information supplied by you in Manuscript Central. You should therefore check that all the information (including author names) is correct as changes will NOT be permitted until the proofs stage.

We look forward to hearing from you and thank you for submitting your manuscript to the European Journal of Immunology.

Yours sincerely,
Karen Chu

on behalf of Prof. Shimon Sakaguchi

Dr. Karen Chu
Editorial Office
European Journal of Immunology
e-mail: ejied@wiley.com
www.eji-journal.eu
